# A Hybrid Indoor Positioning System Based on Visible Light Communication and Bluetooth RSS Trilateration

**DOI:** 10.3390/s23167199

**Published:** 2023-08-16

**Authors:** Lamya Albraheem, Sarah Alawad

**Affiliations:** Information Technology Department, College of Computer and Information Science, King Saud University, Riyadh 145111, Saudi Arabia; 441204669@student.ksu.edu.sa

**Keywords:** VLC, visible light communication, BLE, trilateration, indoor positioning

## Abstract

Indoor positioning has become an attractive research topic because of the drawbacks of the global navigation satellite system (GNSS), which cannot detect accurate locations within indoor areas. Radio-based positioning technologies are one major category of indoor positioning systems. Another major category consists of visible light communication-based solutions, as they have become a revolutionary technology for indoor positioning in recent years. The proposed study intends to make use of both technologies by creating a hybrid indoor positioning system that uses VLC and Bluetooth together. The system first collects the initial location information based on VLC proximity, then collects the strongest Bluetooth signals to determine the receiver’s location using Bluetooth RSS (received signal strength) trilateration. This has been inspired by the fact that there have not been any studies that make use of both technologies with the same positioning algorithm, which can lead to pretty high accuracy of up to 0.03 m.

## 1. Introduction

Localization is a very important asset for many individuals and organizations in today’s world, with numerous location-based applications for both outdoor and indoor communications being put into use. As a result of the diverse coverage and accuracy of these positioning systems, outdoor technology often relies on the Global Positioning System (GPS) or Global Navigation Satellite Systems (GNSS). However, due to the difficulty of the GPS and GNSS-born signals in penetrating walls and ceilings, they are not very reliable for indoor localization. Therefore, indoor localization systems are in development, which consist of a navigation system and tasks involving positioning, planning a feasible route, and guiding users through an indoor route to their desired destination. Indoor localization systems are classified into three categories based on their technology: computer vision-based systems, pedestrian dead reckoning systems (PDR), and communication-based technologies, which include the most commonly used indoor localization systems that exist today [1]. Examples include Wi-Fi, Bluetooth, radio-frequency identification (RFID), visible light communication, and ultra-wideband. These technologies are used for a lot of purposes, such as locating assets within a building or assisting visually impaired individuals [2]. Radio frequency-based technologies are popular for indoor localization but face some challenges when employed, such as high power consumption, security concerns, and low throughput [3].

Furthermore, positioning systems that utilize light sources have gained traction due to the great advantages they offer, such as high bandwidth, high security, low power consumption, a long lifetime, the ability to cover RF-restricted areas, and low cost [2]. These advantages have created the chance for visible light communication (VLC) to be a critical technology for use in indoor positioning systems [2]. VLC systems have seen reasonable advancements, such as the light fidelity (Li-Fi) technology, a VLC technology that fundamentally uses light-emitting diode (LED) bulbs for illumination and data transmission [4]. As mentioned, visible light-based systems offer great potential but still have some limitations, such as covering a relatively small area for each LED, light signal interference affecting signal strength, and connectivity loss due to obstructions [5]. Therefore, some research suggests combining VLC with radio-frequency-based technologies to overcome the drawbacks of using either of these technologies alone [4].

Different indoor positioning technologies use different positioning techniques based on their study goals and requirements. These techniques vary between range-based approaches, range-free approaches, and fingerprinting [6]. Each of these techniques calculates the location of the receiver differently. Range-based approaches provide high accuracy and utilize the geometric properties of triangles to calculate the location; these calculations can be conducted either through trilateration or triangulation [6]. In the trilateration technique, the distance is calculated through either received signal strength (RSS), time of arrival (TOA), or time difference of arrival (TDOA). A range-free approach, such as proximity, provides estimated location information [1]. Lastly, the fingerprinting approach compares the characteristics of the received signal with the previously collected fingerprint map of space, which requires complex computation [6].

As mentioned earlier, localization techniques such as GPS and GNSS are not able to detect locations within a building, which has created a gap that has since been filled with research on many technologies that aim for indoor localization [7]. However, accurate indoor localization is still a work in progress, especially with the rise of VLC in recent years as a new indoor localization technology that has proven to be a cheap and accurate localization method. A lot of research has been dedicated to this new technology, and many studies have explored different methodologies for using VLC [6]. However, after conducting the literature review, it was found that using VLC systems in collaboration with radio-frequency-based systems is a new and interesting area within indoor positioning that needs to be further exploited [2]. One proposed model suggests using a VLC system with Bluetooth [8]. The proposed model used VLC fingerprinting with a Bluetooth spring model; the system achieved high accuracy, but the fingerprinting technique needs high complexity to collect all the illumination information from the LEDs in the database, which can cause positioning delays. In addition, there is the potential challenge of the RSS information of LEDs being affected by reflection, diffraction, or overlapping in illumination [4]. The research concludes that using VLC localization based on proximity can achieve accurate positioning because the light signals are isolated within the room area, which means signals cannot be picked up through walls, unlike RF signals, which initially give accurate results because of the limited receivable area of LEDs. Meaning that a set LED with a known position and known identifier can only cover a small area; therefore, collecting the information of the location of the receiver in proximity to this LED gives an error margin within the coverage area of the LED [9]. Whereas using VLC localization based on RSS information from the light signal can be affected by problems mentioned earlier, such as reflection. Therefore, using the RSS information of an RF-based system such as Bluetooth can help collect the necessary RSS information for the system to calculate the receiver’s location. This is the main motivation behind this research, in which we propose the question: “Does using a hybrid of VLC proximity and Bluetooth using RSS-based trilateration produce accurate results for indoor positioning?” Our aim is that our paper will be a step closer to exploring the area of hybrid indoor positioning systems.

The aim of this paper is to discuss various approaches to Indoor positioning technologies and to propose a hybrid indoor positioning system that utilizes VLC with Bluetooth to locate a receiver via trilateration. This research aims to bridge a gap within indoor positioning systems by introducing a novel system that uses a hybrid of proximity-based VLC and Bluetooth RSS trilateration to localize the receiver. To achieve this goal, there are multiple objectives that need to be met, which are:

1. To investigate relevant studies that utilize hybrid technology for indoor localization;
2. To develop a novel system that uses a hybrid of a proximity-based VLC positioning system and a Bluetooth positioning system that uses RSS-based trilateration for localization;
3. To conduct an experiment to test and evaluate the accuracy of the system.

The remainder of this paper is structured as follows: Section 2 presents a background on light fidelity (Li-Fi) technology, including its architecture and modulation techniques. In Section 3, the literature review focuses on previous studies carried out using indoor localization technologies such as Wi-Fi localization, radio-frequency identification, Bluetooth, and VLC systems. Section 4 presents the proposed methodology, followed by details of the experiment and a discussion of the results in Section 5 and Section 6. In Section 7, an evaluation of the hybrid method is presented. Finally, Section 8 presents the conclusion and directions for future work.

## 2. Light Fidelity

Li-Fi was introduced by professor Harald Haas in 2011 as a wireless communication technology that utilizes visible light instead of radio frequencies for high-speed data communication [10]. Radio frequencies have a smaller range of up to 300 GHz compared to the optical frequency range, which starts at 300 GHz and goes up to 30 Peta Hertz, including infrared, visible, and ultraviolet bands [11]. Li-Fi can overcome the bandwidth limitations of technologies that use radio frequencies, such as Bluetooth and Wi-Fi. However, Li-Fi should be considered a complimentary solution, not just a replacement, for these technologies.

### 2.1. Li-Fi Architecture

The main components of a Li-Fi system are a transmitter (light source) of a light-emitting diode (LED) bulb and a receiver, usually a photodiode or a dongle, for bi-directional data communication.

#### 2.1.1. Transmitter (LED)

The LED bulb is a semiconductor device that emits light when an electric current passes through it. In Li-Fi, the LED works as a data transmission medium [12]. Each LED serves as an access point or optical base since it is driven by a Li-Fi modem or chip. The flickering of the light source acts as a binary signal that is picked up by the receiver and translated into data [10].

#### 2.1.2. Receiver

The receiver is usually a photodiode, a dongle, or a camera used to receive the light signal from the transmitter. The received signal is converted into binary using a signal demodulator. The demodulated signal is then used by the localization algorithm to determine the location [3].

### 2.2. Li-Fi Modulation Techniques

The common modulation techniques used for Li-Fi systems can be classified into two categories: single carrier and multiple carrier modulation. Single carrier modulation techniques such as on–off keying (OOK) and pulse position modulation (PPM) are the most common and are easier to apply in low-speed and low-complexity data transfer applications, such as localization systems [5]. Such modulation techniques usually work by the LED light being rapidly modulated, so that with human-level processing, it is not possible to detect the change in the state of the light [13]. OOK is a well-known and simple modulation scheme, and it offers a nice trade-off between system performance and implementation complexity [14]. In OOK modulation, a bit “1” is represented by an optical pulse, while a bit “0” is represented by the absence of an optical pulse. OOK can also work by varying the intensity, less than and greater than the standard intensity, as a transmission technique for bits “0” and “1”. Using this as a form of modulation appears effective in Li-Fi as it reduces the time for a status change between light intensities compared to switching the LED on and off [13].

## 3. Literature Review

This section focuses on discussing previous studies on communication-based indoor localization technologies. First, studies about indoor localization technologies are presented. Studies of radio frequency-based indoor positioning are presented as follows: Wi-Fi localization is discussed, followed by studies about radio-frequency identification and Bluetooth positioning. Studies about Visible Light Communication systems using different position estimation techniques are then discussed. This is followed by studies that use hybrid RF technologies with VLC. Lastly, a discussion and comparison of the technologies are presented.

### 3.1. Radio Frequency-Based Indoor Positioning Systems

Wi-Fi is one of the most commonly used technologies for indoor localization, with many studies combining it with received signal strength-based techniques or fingerprinting. In [1], Kunhoth considered a Wi-Fi-based indoor navigation system. It was introduced because of the fluctuations in RSS, which caused positioning accuracy issues. A fingerprint spatial gradient (FSG) was introduced to overcome this problem. Kunhoth also noted that in the last 10 years, many machine learning algorithms, such as the support vector machine (SVM), k-nearest neighbor (KNN), and neural networks, have been used for pattern matching in radio fingerprint-based indoor localization methods [1]. Radio-frequency identification (RFID) technology is a popular technology for indoor localization and can be integrated into hybrid indoor positioning systems. Bouet et al. [15] explored RFID as one of the communication-oriented technologies for indoor localization. An RFID tag and RFID reader are needed for an RFID system to work. While there are both passive and active RFID tags, RFID deployed indoors can use passive tags, as an external source of power is not needed. For RFID to estimate the position in indoor positioning, it invokes range-based approaches such as AOA, RSS, TOA, and TDOA. Unlike other methods, RSS delivers an estimation of the position of the user even in non-line-of-sight contexts. Range-based location estimation is needed with RFID to correctly identify a particular object within an indoor positioning system [15]. Bluetooth technology has been a revolution within indoor localization methods, and a lot of research has been dedicated to this technology. In his survey, Kunhoth [1] reported that Bluetooth low-energy beacons function as radio frequency signal sources to help generate user positions and have similar accuracy to Wi-Fi-based systems.

### 3.2. VLC for Indoor Positioning

Recently, Visible Light Communication (VLC) has become a critical technology for navigating in physical spaces as well as for identifying objects in physically confined spaces in individual and industrial-scale applications, offering accurate indoor positioning with a relatively simple system configuration. Here, we considered studies that worked with modified and unmodified light sources to compare the advantages and disadvantages of each. 

In [16], Zhang et al. proposed a VLC indoor localization system that takes into consideration sunlight exposure and uses an RSS-based triangulation method. The transmitters are modified LEDs that do not require synchronization, which means there are no connections needed between the transmitters. Therefore, the system is easy and cheap to deploy in indoor environments. However, because the system has multiple transmitters and one receiver, there is a channel multi-access problem that needs to be solved by using an asynchronous channel multiplexing method called basic framed slotted ALOHA (BFSA) to detect the light source. The system showed promising results, with a precision of 95% within 17.25 cm with direct sunlight exposure and a precision of 95% within 11.2 cm with indirect sunlight exposure. 

A visible light localization system is presented by Li et al. in [17]. The system has two parts: an LED bulb and a receiving device, such as a smartphone. These parts consist of functional modules that work to achieve three key technical components: light beaconing, distance estimation, and localization. The light beaconing component works by using modified LED bulbs to broadcast the location to the receiver. The LED uses a binary frequency shift keying (BFSK) modulation module to encode the messages that are later demodulated at the receiver side. Meanwhile, the distance estimation component on the receiver side decodes the information embedded in the light beacons from multiple light sources and measures their RSS. The localization component locates the receiver using trilateration or multilateration, depending on the number of perceived light sources. This paper proves the great potential of using visual light for high-accuracy indoor localization.

Gu et al. [18] presented an indoor localization system using VLC where LEDs are employed as transmitters and photodiodes are used as receivers that obtain the RSS information from the transmitters based on a trilateration technique. The proposed system design consists of a room with at least four modified LED bulbs installed in a square shape on the ceiling and a receiver that is installed on a mobile device. On–off keying (OOK) is used to modulate the LEDs. The receiver obtains information from all four LED transmitters, which creates a channel multiaccess problem that is addressed by using an asynchronous protocol called a basic framed slotted ALOHA (BFSA) as a channel access method that enables asynchronous transmissions. After the signals are received, an RSS-based trilateration algorithm is applied to detect the receiver’s location. Then, to realize real-time tracking of the receiver, two filters are applied to the system: a Kalman filter and a sequential importance resampling (SIR) filter. Both improve localization accuracy.

A visible light localization system that uses unmodified light sources is proposed in [19] by Zhao et al. The system, “NaviLight”, uses existing lighting sources as transmitters and treats light intensity as the fingerprint, or “LightPrint”, of the light sources that are used to detect the user’s location. The system is inspired by Wi-Fi-based indoor localization systems, which use the Received Signal Strength Indicator (RSSI) fingerprint to detect the user’s location. However, using light intensity as a fingerprint is more challenging with a VLC system since light intensity is more coarse-grained and ambiguous over space compared to electronic signal strength. Additionally, there is no communication between the source and the receiver, unlike in Wi-Fi systems. Therefore, NaviLight uses a vector of multiple light intensity values in combination with the user’s walk or movement to determine location. Because this system utilizes existing lighting infrastructure, it is considered easy to deploy, low-cost, and of high accuracy. It is adaptive and compatible with various indoor environments. However, light intensity information can be insufficient because the light intensity of one location might be similar to another location, and matching the LightPrints to pre-trained data to find the position can be computationally expensive.

Lastly, in [20], Kuo et al. introduced a positioning method using LED lights and smartphones called “Luxapose”. In this method, the LEDs have been modified to emit optical pulses that contain unique location information that cannot be seen by the human eye. An unmodified smartphone camera captures images that detect the presence of the light source, decode the identifiers and positions, and estimate the smartphone’s position and direction relative to the LED lamp. The experimental results demonstrated that this method is capable of achieving localization errors on the decimeter level and a 3° orientation error when walking under overhead LED lights.

### 3.3. Hybrid Radio Frequency-Based Positioning with VLC

Hybrid positioning systems, as their name suggests, aim to combine two or more systems in order to improve the performance that each system provides alone [21]. As each system has its drawbacks, combining two or more technologies into a hybrid system could achieve promising results in indoor positioning. However, systems that propose using a hybrid of RF technologies with VLC have been understudied. The existing VLC hybrid positioning systems are coupled with vision-based or pedestrian dead reckoning systems [2]. Therefore, there is still room to explore and propose solutions to the shortcomings of the existing systems. For example, a solution for the labor-intensive approach of using Wi-Fi fingerprinting by leveraging the already existing Li-Fi infrastructure was proposed by Huang et al. in [22]. The theoretical system works by deriving the location of the receiver based on the Li-Fi signal identifier and then obtaining the distance between a Wi-Fi access point and the identified Li-Fi lamp using coefficient calibration with the RSSI values of Wi-Fi access points. However, this model has not yet been applied to obtain real-world results, although it shows promise as a solution for the complexity of the fingerprinting approach. Luo et al. [8] proposed an effective and low-cost way to deploy indoor positioning using Bluetooth with VLC. The visible light positioning system estimates the initial position information using fingerprinting, then uses a spring model to correct any positioning errors. There are two types of nodes in the spring model: Mobile devices (MD) and anchor points (AP). These nodes form a Bluetooth network of devices that can communicate with each other, where any unknown node (MD) broadcasts its position to a neighboring MD and known nodes (APs), resulting in the MDs and APs being able to obtain the distance between any pair of MDs and APs. Combining VLC and Bluetooth communication generates a hybrid positioning system that offers better trade-offs than using each of these technologies independently. Lastly, in [9], Ziyan Jia proposed a hybrid positioning system between VLC and a wireless sensor network. The system determines the initial position of the receiver using a LiFi-based proximity method, then uses the RSS of the RF signals from the WSN nodes to determine the specific position in the initial area. Minimum mean square error (MMSE) and maximum likelihood (ML) are used to calculate the accuracy of the RSS estimation. This system was able to keep the position estimation error within 20 cm.

### 3.4. Discussion

Indoor localization technology is a major research topic in many areas and has a variety of different types of applications. Many solutions have been proposed involving indoor localization, including visual impairment aids and navigation, wayfinding systems for indoor areas such as shopping malls, museums, and airports, and indoor positioning for assets in companies and warehouses [1]. However, Rahman et al. [3] identified high power consumption, security concerns, and low throughput as the major challenges of radio frequency-based indoor localization technologies. A comparison with VLC-based systems is presented in Table 1.

RFID and Bluetooth, as indoor positioning techniques, both have low power consumption. However, Bluetooth has a limited coverage range, while RFID’s constraints are the need for an RFID reader, the high response time, and limitations in user ability. Wi-Fi has a high level of power consumption and deployment costs for building databases when fingerprinting is used. VLC-based positioning systems have several advantages. They can be installed inexpensively since they utilize existing lighting systems with very few modifications required. Consequently, implementing VLC increases the chances of identifying objects in physical spaces.

Moreover, after reviewing the research on indoor localization using VLC, it is clear that VLC systems perform better with modified light sources since working with unmodified LEDs requires expensive computation [19]. Pairing a VLC system with a radio-based technology such as Bluetooth achieves higher accuracy than VLC alone (as shown in Table 1) [8]. This was supported by Luo et al. in [4], where they note that integrating VLC systems with wireless technologies such as Wi-Fi or Bluetooth can create a hybrid system that has the advantages of both technologies. Furthermore, position estimation techniques varied from study to study depending on the study goals; some presented certain challenges. For example, using fingerprinting as a positioning technique with light can be complex because of the similarity of light intensity levels between light sources as well as the computational complexity of matching a light intensity to a light map [19]. Computational complexity is a general setback of the fingerprinting technique in almost any system [4]. On the other hand, proximity is the simplest and fastest positioning technique, and it works best if the system needs a rough location, which is helpful in the case of VLC systems because of the limited receivable area of LEDs. When an LED is set in a location, the possible position area of the receiver will be small, creating a relatively accurate position decision for the receiver [2,18]. Triangulation-based positioning has been proven to work well with LED-based systems. However, it is sensitive to the external environment and requires a direct LOS from the source, and it is also not widely used in radio-based systems, which typically have no LOS, such as Wi-Fi and Bluetooth [2].

Lastly, trilateration-based positioning has achieved very accurate results, especially RSS-based trilateration, where the system calculates the location based on the strength of the transmitted signals. This works best with radio-based localization systems because they do not require direct LOS and there is no overlapping of the light intensities, like in VLC-based systems. On the other hand, TOA-based trilateration requires perfect synchronization between the sender and receiver [6].

Positioning with VLC varies in accuracy depending on the technique. Problems like light signal interference can affect the accuracy of an RSS-based approach because of the interference’s effect on the light intensity value [4]. This can be avoided with the integration of the VLC system with radio-based technology. The literature suggests that no studies have used a modified VLC source with Bluetooth using RSS-based trilateration as a positioning technique, which we cover in this paper.

## 4. Proposed Methodology

### 4.1. Indoor Localization System

**Figure 1:**
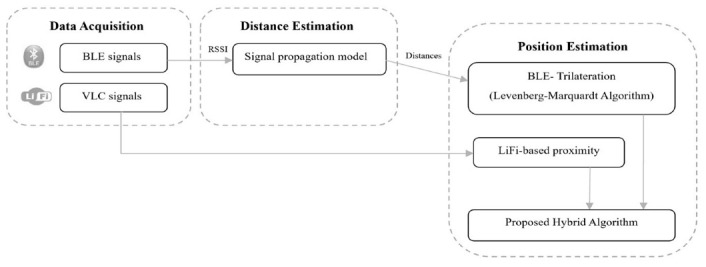
Proposed system architecture.

The aim behind this study was to develop a novel hybrid indoor positioning system that uses VLC with Bluetooth trilateration in order to achieve accurate indoor positioning. This section explains the steps taken to implement a system that used both technologies to achieve high-accuracy indoor positioning. The system consisted of a hybrid between a VLC-based positioning system and a Bluetooth-based positioning system. The system started with data acquisition from the VLC system and the BLE system. The VLC system estimated the location of the receiver based on modulated information sent by the light source, which was then picked up and demodulated at the receiver’s side as a distinct identifier code, and then the Bluetooth system detected the location of the receiver using an RSSI-based trilateration technique. After the results from both systems were calculated, the distance between the VLC and the BLE estimated position was calculated to be used as a fourth triangulation point to calculate the position. The exact steps for building the proposed system are presented below in Figure 1. 

#### 4.1.1. LiFi-Based Proximity

The VLC system consisted of a modified light source (LED light) and a receiver, which was a dongle on the mobile device. Each light source had its own distinct code; this information was modulated at the source, transmitted to the receiver in the modulated format, and then demodulated at the receiver’s side. The receiver’s location was initially estimated in proximity to this light source based on the transmitted information. This location was saved as (x_0_, y_0_) coordinates on the map. The LED code helped to distinguish which floor of the building and which room the light source was in, as well as the location inside the room within a proximate range, which fell within the coverage area of the LED. This initial position was later used to determine the position in collaboration with the Bluetooth-estimated position based on a calculated distance.

#### 4.1.2. Bluetooth Distance Measurement

In order to estimate distance from the RSSI, many models might be used, such as the Log Distance Path Loss model, the International Telecommunication Union (ITU) model, and the empirical model mentioned in [23]. One model takes into consideration how different combinations of transmitters and receivers can affect the results of the RSSI data measurements: the Signal Propagation Model [24]. The Bluetooth system worked by picking up the three strongest Bluetooth signals from different beacons. Every Bluetooth beacon broadcasts its RSSI. The RSSI values were collected to compute the location of the mobile device via a trilateration algorithm. To predict distance from signal strength (RSSI), the Signal Propagation Model was used based on a power regression against a known table of distances/RSSI values. Distance was obtained from the Bluetooth beacons using the formula:

(1) $$d=\alpha \times {\left(\frac{r}{t}\right)\;}^{\beta }+C$$

where *d* is the distance in meters is, *r* is the RSSI measured by the device, and *t* is the reference RSSI at 1 m. *α*, *β*, and *C* are regression coefficients that are derived empirically by using RSSI measurements at multiple distances for different devices. To calculate the device’s location, Algorithm 1 was used.
**Algorithm 1** Distance from Bluetooth using the propagation model Input: RSSI signal.   Output: Distance. Calculate α, β, and C. Calculate Ratio = $\frac{r}{t}$   If Ratio < 1.0,     **calculate** ${d=\left(Ratio\right)}^{10}$   **Else**,     **calculate** $d=\alpha \times {\left(Ratio\right)}^{\beta }+C$   Return d.

#### 4.1.3. Position Estimation via Trilateration

**Figure 2:**
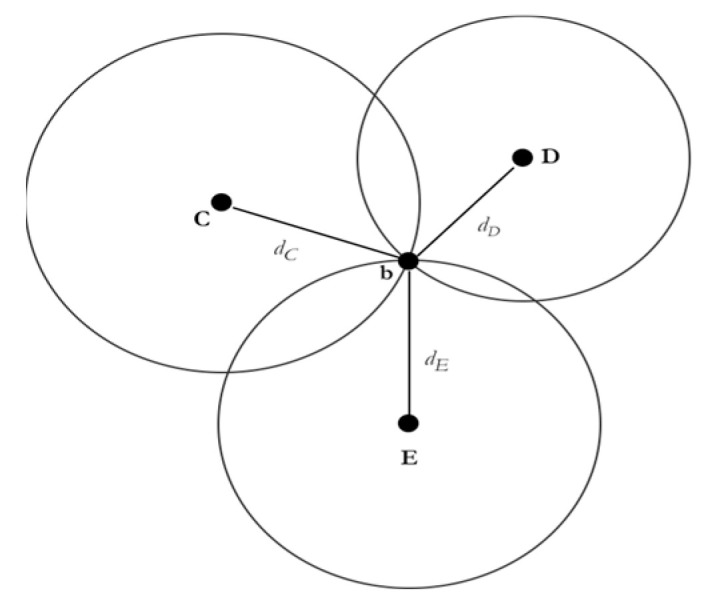
Trilateration function.

The RSSI signals from the Bluetooth beacons were converted into spatial distances, which were used as the radii of circles. The distance from each beacon was calculated. As shown in Figure 2, the trilateration algorithm calculated the coordinates of the intersection of those distances (circles) based on the received signal strength of each signal.

(2) $$d_{C}^{2}={\left(x_{b}-x_{C}\right)}^{2}+{\left(y_{b}-y_{C}\right)}^{2}\;$$

(3) $$d_{D}^{2}={\left(x_{b}-x_{D}\right)}^{2}+{\left(y_{b}-y_{D}\right)}^{2}\;$$

(4) $$d_{E}^{2}={\left(x_{b}-x_{E}\right)}^{2}+{\left(y_{b}-y_{E}\right)}^{2}\;$$

This formula, which is denoted above, is the traditional formula of the trilateration method, where all three circles intersect perfectly at one point. This does not occur in all cases, which means that there is a need for advanced algorithms to approximate the position and obtain better accuracy. An example of this is non-linear optimization algorithms, such as the Levenberg–Marquardt algorithm.

##### Levenberg–Marquardt Algorithm

To improve the accuracy of trilateration in scenarios where there might not be a perfect intersection, algorithms that solve non-linear least squares problems, such as the Levenberg–Marquardt algorithm, can be used. LM interchanges between two minimization methods: the Gauss–Newton and gradient descent methods to solve non-linear least squares problems. It acts more like the Gauss–Newton method when the damping parameter value is smaller and like the gradient descent method when the damping parameter value is larger. The input for this algorithm is a set of n pairs (xi, yi), and the goal is to obtain the parameter p of the model curve f(p) to try and obtain an as small as possible sum of squares of deviations [25]. The simplest way to denote the linear approximation of the function is shown in Equation (5), where p is the parameter vector and J is the Jacobian matrix $\frac{\partial f(p)}{\partial p}$.

(5) $$f\left(p+\delta_{p}\right)\approx f\left(p\right)+J\delta_{p}$$

As with all non-linear optimization methods, LM is iterative, with the parameter vector initialized at $p_{0}$. LM carries out an iterative process to produce a series of vectors, aiming towards a local minimizer $p^{+}$ for the function [25]. This method is a direct interpolation between the Gauss–Newton algorithm and gradient descent, focusing on finding a local minimum rather than a global one.

#### 4.1.4. Hybrid Position Estimation

**Figure 3:**
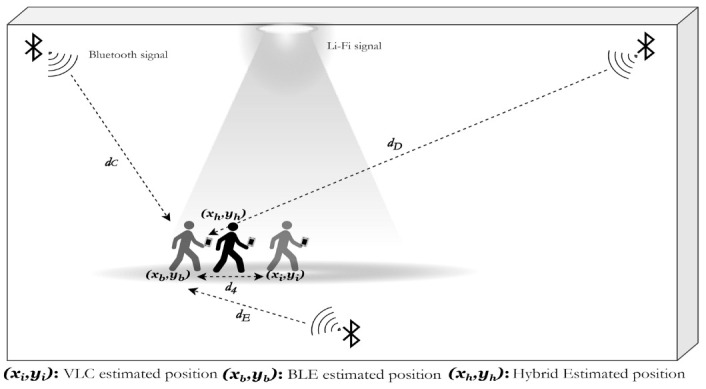
Hybrid system design.

**Figure 4:**
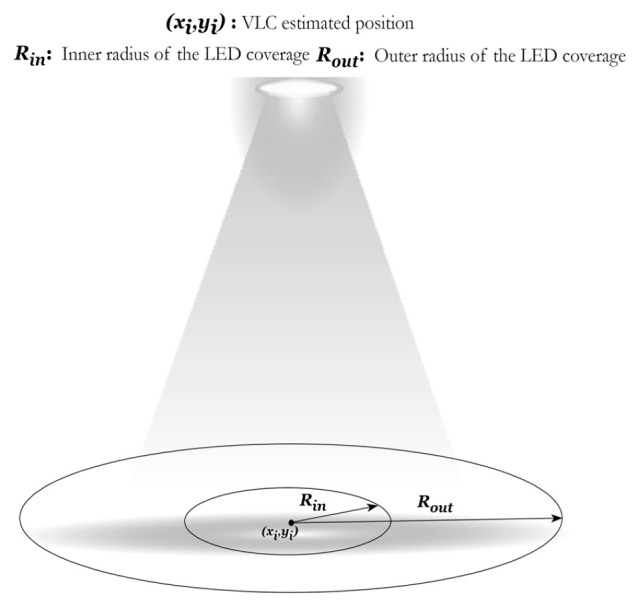
Inner and outer radius of the VLC coverage area.

The methodology of the hybrid system is presented here in detail. As shown in Figure 3, the initial estimated position (x_i_, y_i_) was estimated using VLC, and the (x_b_, y_b_) position was estimated using Bluetooth trilateration. A new position estimation method that used both technologies was created that worked by calculating the distance between (x_i_, y_i_) and (x_b_, y_b_), also known as $d_{4}$ (the 4th distance), since we had three distances from the Bluetooth beacons using the trilateration system. $d_{4}$ was an estimate of the position between the user’s BLE-based position and the center of the LED (x_i_, y_i_). This distance could be used as a notation to the system if the user was closer to the center, the edges, or the outside of the area covered by the LED. When $d_{4}$ denoted that the user was estimated to be closer to the center of the LED, or in other words, the user was inside the inner radius area shown in Figure 4, then $d_{4}$ was used for multilateration to assume a new position (x_m_, y_m_). In the case that $d_{4}$ denoted that the user was inside the area covered by the LED but closer to the edges (or in the outer radius area), then the appropriate action was to take the estimated position from the BLE system. Lastly, when $d_{4}$ denoted that the user was outside the coverage area of the LED even though the LED signal was picked up by the device, the BLE system assumed a position with a bigger error value than the coverage area of the LED, and the user was then assumed to be under the LED and given the value (x_i_, y_i_) of the center of the LED, which gave an error of the coverage area of the LED or less. These scenarios are presented in brief in Table 2 below.

**Figure 5:**
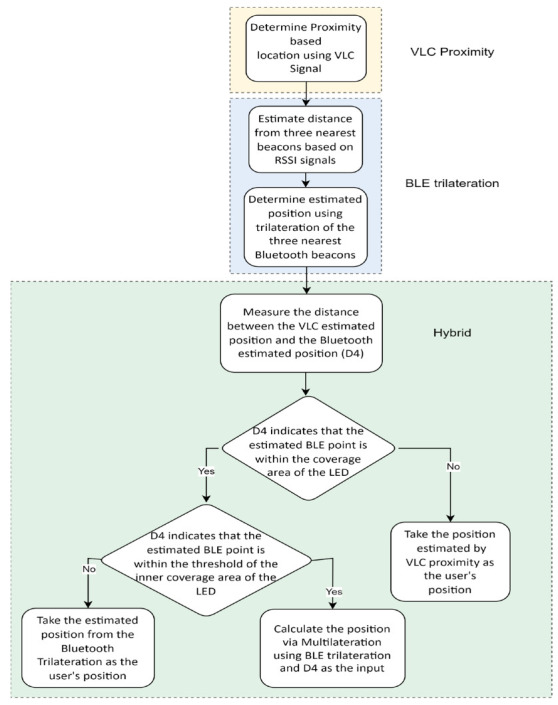
The system methodology flowchart.

As mentioned above, many techniques were considered for the proposed system, such as proximity, trilateration, and multilateration. The steps followed for this system are given in Figure 5, where the flow of the methodology is shown in detail. 

In Equation (6), we present the scenarios in a mathematical format, where $d_{4}$ represents the distance between the BLE-estimated position and the LED, where (x_h_, y_h_) is the hybrid position, (x_i_, y_i_) is the VLC-estimated position, (x_b_, y_b_) represents the position estimated by BLE, and (x_m_, y_m_) is the position estimated by multilateration.

(6) $$f\left(x_{h},y_{h}\right)=\left\{\begin{array}{c} \left(x_{m},y_{m}\right),\;d_{4}\leq R_{in}\;\\ \left(x_{b},y_{b}\right),\;R_{in}<d_{4}\leq R_{out}\\ \left(x_{i},y_{i}\right),\;d_{4}>R_{out} \end{array}\right.$$

To estimate the threshold for the inner radius space, we used the formula proposed by Athanasios et al. [26], which they used to determine an inner radius for their LED coverage area. For this experiment, we wanted a smaller inner radius since our LEDs were at a lower height, so we decided to take a smaller value for the denominator to minimize the inner radius, according to the experiment parameters. Our modified formula is shown below:

(7) $$R_{in}=\frac{\sqrt{A}}{6}$$

where *R_in_* is the inner radius of the LED and *A* is the coverage area of the LED, which is calculated using: $A=\pi r^{2}$.

To summarize, the hybrid algorithm is presented in sequence below in Algorithm 2.
**Algorithm 2** Hybrid Algorithm**Input**: VLC signal, RSSI signals, R_in_, R_out_ **Output**: position ($x_{h},y_{h}$)1:$\left(x_{i},y_{i}\right)$= Determine the proximity-based location using VLC signal2:Get the three nearest RSSI signals from beacons3:Estimate distance from the three nearest beacons based on RSSI signals using power regression:4:$d_{C}=\alpha \times {\left(\frac{r}{t}\right)}^{\beta }+C$5:$d_{D}=\alpha \times {\left(\frac{r}{t}\right)}^{\beta }+C$6:$d_{E}=\alpha \times {\left(\frac{r}{t}\right)}^{\beta }+C$7:Determine estimated position using trilateration of the three nearest Bluetooth beacons using the Levenberg–Marquardt algorithm8:$x_{b},y_{b}=Trilateration_{Levenberg-Marquardt}\left(d_{C},d_{D},d_{E}\right)$9:Measure distance between the VLC-estimated position and Bluetooth-estimated position: $d_{4}$ = $\sqrt{{\left(y_{b}-y_{i}\right)}^{2}+{\left(x_{b}-x_{i}\right)}^{2}}$10:If $d_{4}$ > R_out_11:$x_{h},y_{h}=\left(x_{i},y_{i}\right)$12:**Else if** R_in_ < $d_{4}$ ≤ R_out_13:$x_{h},y_{h}=\left(x_{b},y_{b}\right)$14:Else15:$\left(x_{m},y_{m}\right)=Multiateration_{Levenberg-Marquardt}\left(d_{C},d_{D},d_{E},d_{4}\right)$16:$x_{h},y_{h}=\left(x_{m},y_{m}\right)$

### 4.2. Performance Metrics of the Proposed System

#### 4.2.1. Accuracy

In our research, accuracy was defined as the level of correctness when determining the location of the receiver via the positioning system versus the actual location of the receiver. To determine the level of accuracy, we used Euclidean distance error, which is a widely used metric for determining the accuracy of indoor positioning systems and works typically by comparing the predicted location with the actual location of the user’s device using the Euclidean distance error formula [27].

(8) $$d\left(x,y\right)=\sqrt{\sum_{i=1}^{n}{\left(y_{i}-x_{i}\right)}^{2}}$$

As shown in Equation (8), to determine the distance error, the predicted location of the user’s device was compared to the actual location, and the distance between them was calculated as the square root of the sum of the squared differences between the predicted and actual coordinates. In the case of our research, the dimensions were the (x, y) coordinates of the user’s device. This formula provided an accurate measure of the difference between the predicted and actual locations of the user’s device. A smaller distance error indicated a more accurate position, as it meant the predicted location was closer to the actual location. Conversely, a larger distance error indicated a less accurate position.

The Euclidean distance error is a valuable metric for evaluating the accuracy of indoor positioning systems. It provides a quantitative measure of the difference between the predicted and actual locations of a user’s device, which is essential for evaluating the performance of the positioning system [1].

In addition to calculating the error for each individual position using the Euclidean distance error, we took the mean value, which represents the average error, as well as the minimum and maximum values for error and the total error in distance units. Finally, we calculated the standard deviation and confidence interval for the samples.

Another method of error calculation in the literature was the root mean square error (RMSE), which we calculated in this experiment for evaluation and comparison purposes. The RMSE is calculated by taking the square root of the mean of the squared differences between the predicted and actual values. It is used to evaluate the accuracy of a prediction model by measuring the average magnitude of the error [28].

(9) $$RMSE=\sqrt{\frac{1}{n}\sum_{i=1}^{n}{\left[(x_{a}-x_{p}\right)}^{2}+{\left(y_{a}-y_{p}\right)}^{2}]}$$

where n is the total number of observations, x_p_ and y_p_ are the predicted values for the position, and x_a_ and y_a_ are the actual values for the position.

#### 4.2.2. Capability of Producing an Optimal Solution

The proposed hybrid was intended to select either VLC proximity or BLE trilateration or combine both technologies using multilateration based on the value of $d_{4}$. In addition to evaluating accuracy, we must also ensure that the hybrid system performs in an optimal manner. We achieved this by measuring the ability of the hybrid system to obtain the optimal method to find an accurate position and evaluating the individual cases to see if the hybrid system selected the technology with the lowest error in every case. This measure is represented by the number of cases where the system performed optimally and is given as a percentage.

(10) $$optimal\;solution\;percentage=\frac{optimal\;positions}{all\;positions}\times 100$$

## 5. Experimental Studies

This chapter presents the experimental studies conducted to evaluate the performance of the proposed system in a specific experiment testbed. The general design of the experiments is presented first in Section 5.1, then the hardware used in the experiments is described in detail in Section 5.2, while Section 5.3 focuses on the software used for the experiments.

### 5.1. Experiment Testbed

**Figure 6:**
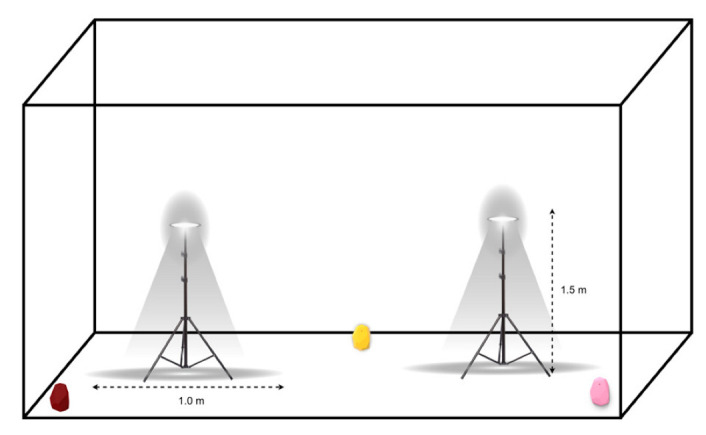
Experiment design.

In this experiment, two test spaces were used in order to evaluate the performance of Bluetooth positioning in both smaller and larger areas. Both spaces had almost no interference for either Bluetooth or VLC. Based on the tools available as well as the layout of the available spaces, while trying to ensure feasibility and practicality, we designed the experiment as follows: Two LEDs at a height of 1.5 m covered a diameter of about 1 m and an area of about 0.79 m^2^. The LEDs did not intersect in any scenario since they were placed more than 1 m apart, with three BLE beacons in fixed placements. As shown in Figure 6, the use of these fixed placements allowed for a more controlled testing environment, ensuring that any variations in the results could be attributed to factors such as the size of the testing environment and not obstacles or changes in equipment. Overall, the similarity in design between the two testing environments provided a comprehensive platform for evaluating the performance of the positioning system, particularly in scenarios where the size of the testing environment varied.

#### 5.1.1. First Environment Parameters

**Figure 7:**
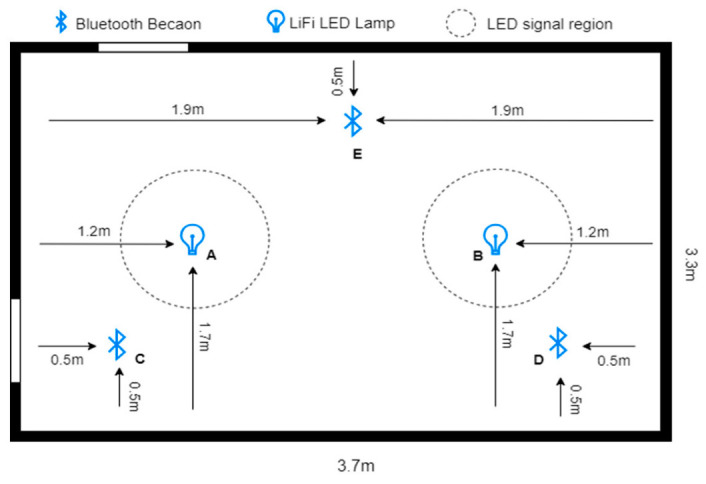
Two-dimensional design of the first space.

In the first space, two light fixtures labeled A and B were set up to send VLC signals, and three Bluetooth beacons were distributed in the 3.7 × 3.3 × 3 m room. The three Bluetooth beacons, labeled C, D, and E, were placed in fixed locations within the room. Similarly, the two LEDs were also placed in fixed locations. This setup allowed for reliable experimental conditions and data collection. To act as the user’s device in the experiment, an Android mobile phone named River 1, provided by the Saudi company Azom [29], was used. The dimensions of the area and the distribution of the equipment in the first environment are shown in Figure 7. The parameters of the experiment are displayed in Table 3. A detailed explanation of the hardware and software specifications used in the experiment is given in the following sections: By meticulously designing the experiment space with specific hardware and software components, we were able to create a controlled environment for the experiments and obtain reliable data.

#### 5.1.2. Second Environment Parameters

**Figure 8:**
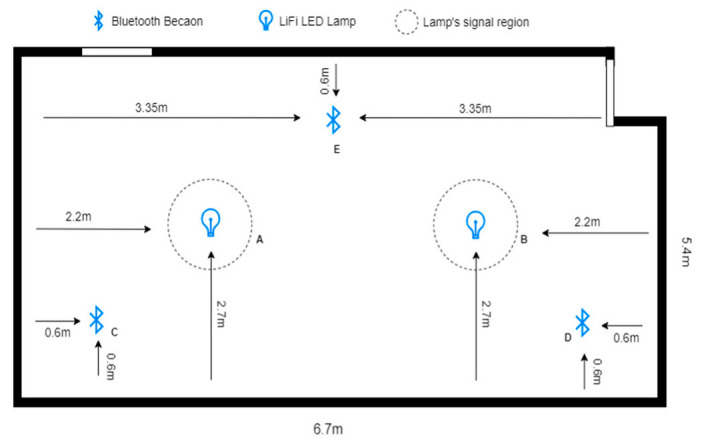
Two-dimensional design of the second space.

The second experiment space was similar to the first, with the same two LEDs and three Bluetooth beacons. However, the dimensions of this room were slightly different, measuring 6.7 × 5.4 × 3 m. Like in the first experiment space, the two LEDs in the second room were fixed in their locations at the same height as in the first environment. This allowed for consistent lighting conditions throughout the room during the experiments. In addition, the three Bluetooth beacons in the second room were also fixed in their locations. The locations of these beacons are shown in Table 4. Overall, the second experiment space was designed to be as similar as possible to the first room, with the same equipment and setup. This allowed for greater consistency and accuracy in the experimental results and made it easier to compare the data collected in the two spaces. The mapping of the area, space, and placement of equipment in the second testing environment is shown in Figure 8.

### 5.2. Hardware

#### 5.2.1. LED Lamps

**Figure 9:**
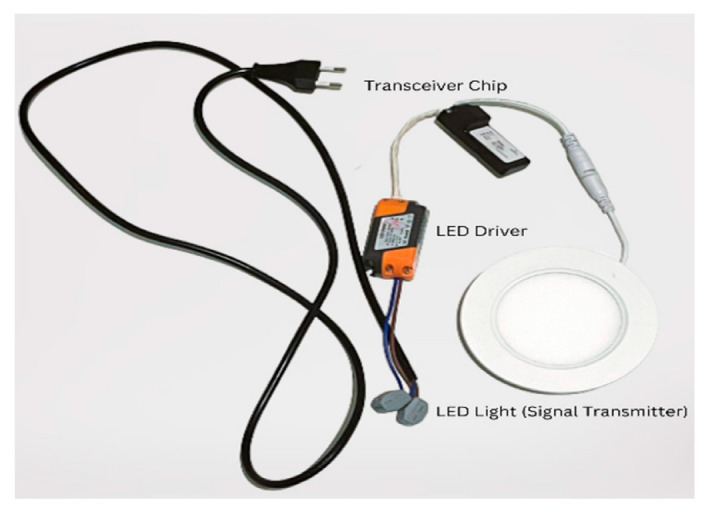
GeoLiFi development kit.

**Figure 10:**
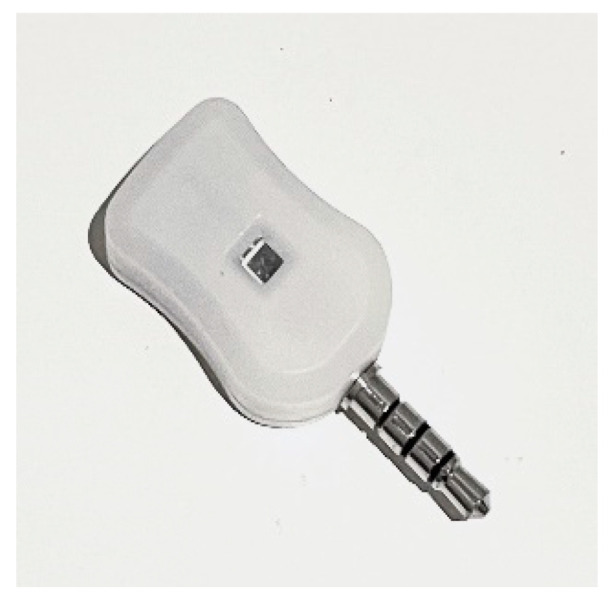
GeoLiFi dongle.

For the VLC source, GEOLiFi LED kits were used to carry out the experiment. The pre-modulated kit consisted of an LED Lamp powered by an LED driver that uses a router to send the LiFi signals through the lamp and a dongle to receive the light signals on the mobile device. The kit was compatible with mobile cameras using an SDK for iOS, but as this experiment was carried out with an Android, the dongle was used alongside the appropriate software. This kit was powered by Oledcomm [30], which is a French-based company that offers a large range of LiFi solutions, such as modems, dongles, and dedicated SDKs. Device specifications are given in Table 5 and shown in Figure 9 and Figure 10.

#### 5.2.2. Bluetooth Beacons

Bluetooth low energy, also known as BLE, is a form of wireless communication technology that became popular after the introduction of the Bluetooth 4.0 BLE Standard in 2009. BLE is widely used in various indoor environments. The specifications of the standard have allowed more advanced use of the technology for localization purposes by introducing a new type of device called “Bluetooth beacons”. Unlike devices that used the previous standards, the new devices have the option of transmitting at set intervals, which contributes significantly to the energy efficiency of the system. The positioning algorithms are mainly divided into two categories: the received signal strength indication (RSSI) distance method and wireless fingerprint positioning technology. Generally, the RSSI distance method acquires the received signal strength (RSS) from the Bluetooth anchor point to determine the distance-loss model and then estimates the user’s position through different algorithms, such as trilateration. Estimote Inc. [31] is a company that provides different types of beacons, including the BLE beacons that were used in this experiment. Three location beacons were used in this experiment with a broadcasting power of 4 dBm, a 70 m range, and a 200 ms advertising interval. 

The location beacons provided by Estimote were designed specifically for indoor location tracking and positioning. They incorporate a range of technical specifications that enable them to deliver precise and reliable indoor positioning, such as having a range of up to 70 m in open spaces, although the range may be shorter in indoor environments with obstacles such as walls or furniture. They have a broadcasting power of 4 dBm, which allows for strong signal transmission without compromising the battery life too much. In addition, their advertising interval provides a balance between accuracy and battery life. Finally, Estimote Location Beacons support both the iBeacon and Eddystone protocols, which are the most commonly used protocols for beacon technology. These specifications are given in Table 6 below.

### 5.3. Software

#### 5.3.1. LiFi Proximity

**Figure 11:**
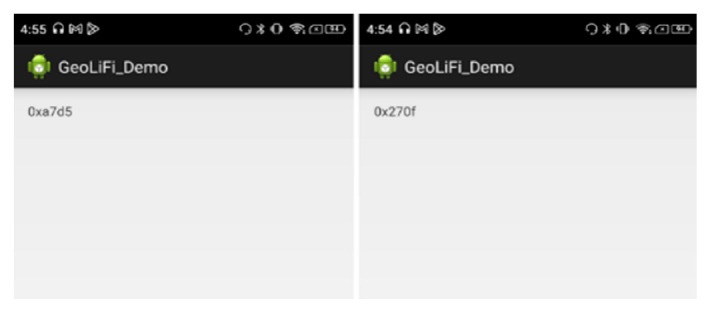
When the signal is detected.

**Figure 12:**
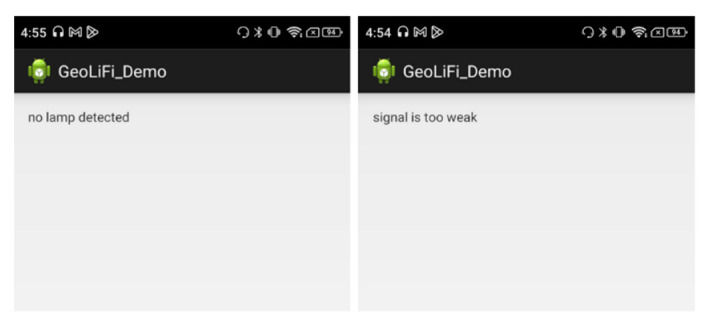
No signal is detected.

The GEOLiFi kit comes with a software development kit that is programmed to be able to read the LiFi signal through the dongle and identify the ID of the lamp whenever the device is within the range of the light signal coverage area. The ID is transmitted using Li-Fi and received through a dongle to be demodulated and then displayed on the device, as shown in Figure 11. If the signal is too weak or if the device is out of the LED signal range, the appropriate message is displayed on the screen, as shown in Figure 12.

#### 5.3.2. Bluetooth Distance

**Figure 13:**
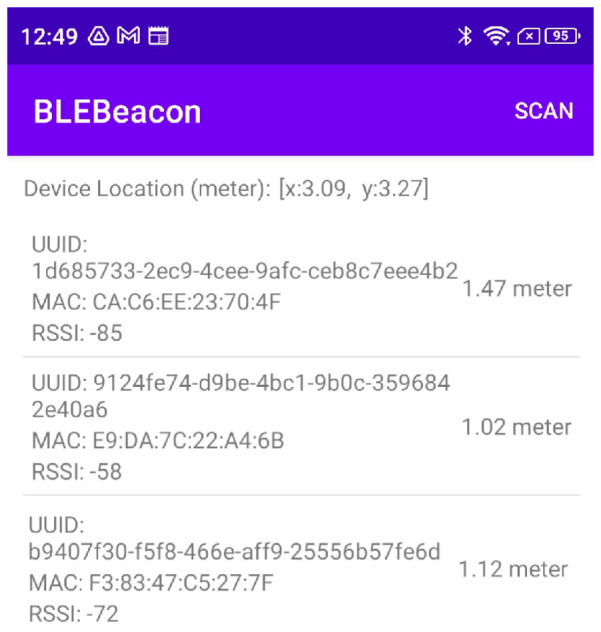
Trilateration Bluetooth.

Since Estimote beacons support the iBeacon protocol, Estimote Inc. has released an SDK for iOS devices to read distance from their distance beacons. For an Android device, we built a simple Android application using the AltBeacon Android library [32], which allowed Android devices to use beacons much like iOS devices do. Each beacon’s distance was measured based on their RSSI using the power regression model mentioned in Equation (1). An overview of the application is shown in Figure 13.

#### 5.3.3. Trilateration

When three Bluetooth distances were obtained, the location was calculated using the trilateration algorithm provided by LemmingApex’s Trilateration Library [24]. This library has been extensively used for computing the user’s position using trilateration from the distances of multiple Bluetooth signals. We calculated the estimated position using a non-linear least squares optimization algorithm. After taking this information, necessary calculations using LM were performed, and the estimated position is displayed as the position (x, y) in Figure 13.

#### 5.3.4. The Hybrid Technique

Hybrid positioning systems, as their name suggests, are meant to be able to combine two or more systems in order to improve the performance that each system provides alone. To achieve higher accuracy in the hybrid technique, a threshold was introduced to select the points closer to the center of the LED for the multilateration. This threshold was evaluated by calculating the coverage area of the light source using $A=\pi r^{2}$. Then, we set up the threshold value for the smaller radius to be accepted for $d_{4}$, as shown in Figure 5. The value of the inner radius was calculated for this experiment as follows:

The coverage area of the LED is $A=\pi 0.5^{2}$ = 0.7854 m^2^.

The inner radius based on Equation (7) is $r_{inner}=\frac{\sqrt{A}}{6}$ = $\frac{\sqrt{0.7854}}{6}$ = $\frac{0.8862}{6}$ = 0.15 m.

This means that the threshold where $d_{4}$ was used in multilateration was 0.15 m. When the value of $d_{4}$ was less than 0.15, the user’s position was calculated based on multilateration. Any value of $d_{4}$ that was larger than 0.15 but less than 0.50 meant the user’s position was based on the Bluetooth system. Lastly, whenever the value of $d_{4}$ was greater than 0.50, it meant the user’s position was based on the VLC system. The cases for the used equipment are shown in the following equation:

(11) $$\left(x_{h},\;y_{h}\right)=\left\{\begin{array}{c} \left(x_{m},\;y_{m}\right),\;d_{4}\leq 0.15\\ \left(x_{b},\;y_{b}\right),\;0.15<d_{4}\leq 0.50\\ \left(x_{i},\;y_{i}\right),\;\;d_{4}>0.50 \end{array}\right.$$

## 6. Results and Discussion

### 6.1. First Experiment Environment

**Figure 14:**
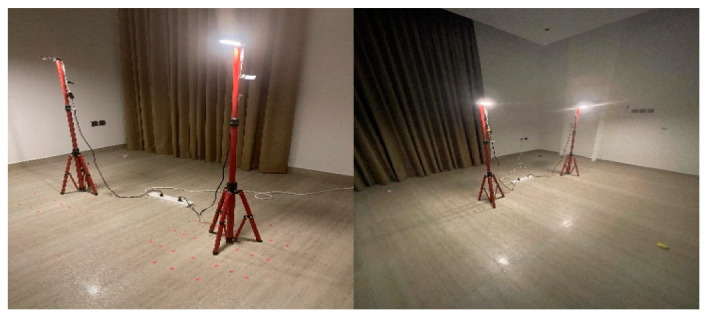
First experiment environment.

The experiment was carried out initially in a smaller testing area, as shown in Figure 14. The dimensions of the room were 3.7 × 3.3 × 3 m^3^, and over 50 estimations were taken in variable positions around each light fixture. In each estimation, the initial position was detected using VLC, and after that, the other position from the Bluetooth system was detected via trilateration. To ensure accuracy against variations in the Bluetooth signals caused by any disturbance, such as a change in height or direction or signal interference, 10 Bluetooth readings were taken for each position and averaged. This value was considered to be the BLE reading for this position. After that, the position between the VLC center and the estimated BLE position was calculated to detect if the user was standing in close proximity to the center of the LED or the edges. This value, which was called $d_{4}$, or fourth distance, was used as a fourth distance value in the multilateration, where the LED position was the center and $d_{4}$ was the radius. 

The experiment was carried out in 52 positions around each LED. These positions were marked on the floor in order to ensure the user was standing in the same position for each reading. Ten readings for each position were taken and averaged to create a BLE estimated position, for a total of 520 readings. Then, the error of the distance in Bluetooth, VLC, and hybrid systems was calculated using the Euclidian distance error mentioned in Section 4 of Chapter 4.

Table 7 shows the error measured in Euclidian distance to estimate the position accuracy using VLC technology, Bluetooth trilateration, and the hybrid technique. 

From Table 7, we can see that the majority of the cases chose the positions estimated by Bluetooth based on the value of $d_{4}$. Only 4 values out of 26 under this position were either in the inner radius or outside the area based on $d_{4}.$ In position 5, the hybrid method favored the multilateration approach, which proved to be better than both BLE and VLC. However, when multilateration was used for positions 8 and 9, it did not achieve the lowest error rate, even though they had low errors of 0.19 and 0.16, respectively. Additionally, in position 15, the VLC approach was used because the value of $d_{4}$ was larger than the threshold and it achieved a lower error than BLE and multilateration. Lastly, positions 6, 14, 18, 21, and 26 favored the BLE estimated position, despite it not being the most accurate position in these cases. Again, this was because the value of $d_{4}$ depends on the accuracy of BLE and does not always reflect an accurate estimation of the position between the device and the LED. Regardless, the optimal solution percentage for the hybrid algorithm under the first LED was 73.1%, where 19 out of 26 positions achieved the best possible accuracy.

**Figure 15:**
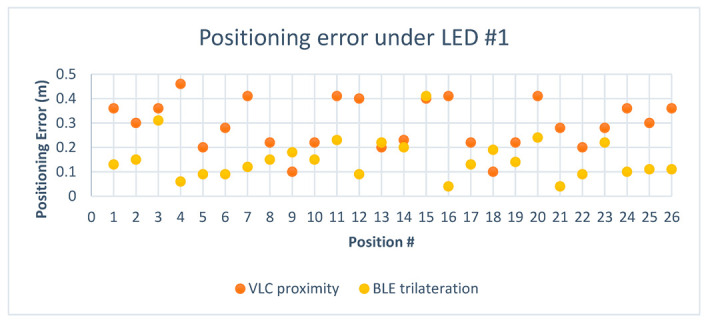
Positioning error under LED #1 for BLE and VLC in the first environment.

To visualize the positioning errors presented in Table 7 for VLC proximity vs. BLE trilateration, the chart in Figure 15 displays a comparison of the error in distance for positions under the first LED using both technologies. The results indicated that BLE trilateration achieved lower error rates than VLC under the first LED in the first environment. This observation can be attributed to the size of the testing area, suggesting that BLE technology may be better suited for smaller spaces.

**Figure 16:**
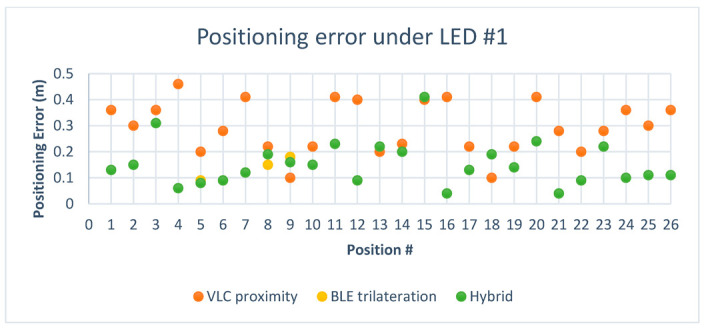
Positioning error under LED #1 for all technologies in the first environment.

After adding the results obtained from the hybrid system to the chart, we can see in Figure 16, where the green points represent the hybrid system, that it leaned towards the Bluetooth system in regard to positioning since the Bluetooth system performed very well in this area.

Similar to the first LED, we estimated 26 positions under the second LED using the same method. The results from this test contributed to a comprehensive overview of the positioning accuracy of each technology. The experiment involved testing various positions around each LED within its coverage area. To qualify for testing in this experiment, a position had to pick up at least three Bluetooth signals as well as the LED ID.

**Figure 17:**
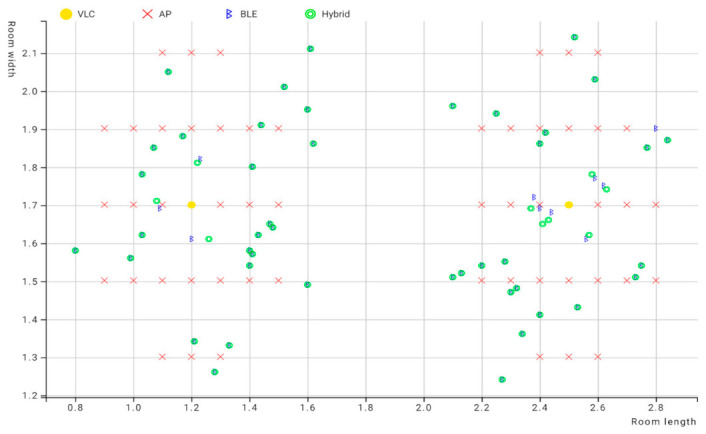
Actual vs. detected positions in the first experiment.

Table 7 presents the chosen testing positions. Once readings were taken for each position, the results obtained from the LED system and the BLE system were compared. After calculating $d_{4}$ for each position and comparing it against the inner radius threshold, the hybrid technique was implemented. When a position was within the outer radius area, the value of the BLE position was used. However, when a position was estimated to be within the inner radius area, multilateration was used. The positions estimated using this technique are depicted in Figure 17.

As demonstrated in Figure 17, the hybrid positions were closely aligned to the BLE estimated positions because of the small difference in error margin between the two technologies, since BLE achieved a low error margin in this small environment due to the small distance between the beacons and the user’s device, which meant the BLE estimated positions were less affected by signal disturbance. The position of the LEDs in this area was also closer to the beacon positions, leading to the majority of the BLE estimated positions giving a value of $d_{4}$ within the coverage area of the LED. After applying the scenarios from Equation (6), the system leaned towards the use of BLE technology in this experiment.

**Figure 18:**
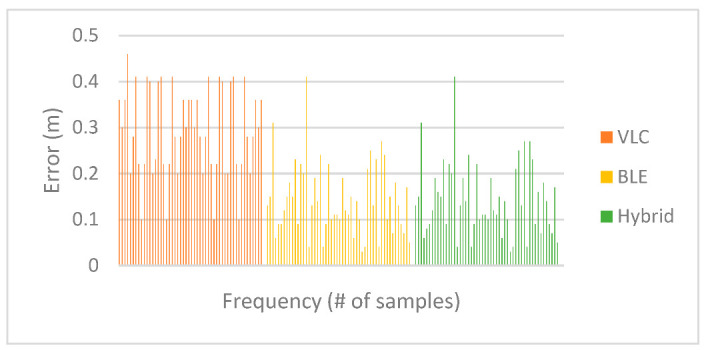
Error using different technologies in the first experiment.

Figure 18 displays the Euclidean distance error values for each technology used in the experiment. As the experiment was carried out in a small area, the Bluetooth system exhibited a high level of accuracy, leading to the hybrid method achieving a high degree of accuracy. As previously mentioned, the results obtained from the VLC system were reasonably accurate. However, given the exceptional performance exhibited by the BLE system within the limited testing space, it was favored by the hybrid system for position estimation based on the values of $d_{4}$. In regards to the optimal solution percentage for the hybrid algorithm in this environment, 14 out of 52 total positions did not achieve optimal positioning, which put the optimal solution percentage for the hybrid system at 73.1%. The individual cases were discussed previously.

Table 8 presents a comparison of the Euclidean Distance Error values for the first space, including metrics such as the mean, minimum, maximum, total distance, standard deviation, and 95% confidence interval.

From Table 8, it is noted that the mean distance estimated by VLC was 0.28 m, by BLE was 0.15 m, and by hybrid was 0.14 m. This means that in this experiment, on average, VLC estimated the distance to be greater than the BLE and hybrid methods. The total distance estimated by VLC was 14.73 m, by BLE was 7.85 m, and by hybrid was 7.46 m. This indicates that VLC measured the longest total distance in the smaller area. In addition, the standard deviation of distance measurements by VLC was 0.10 m, by BLE was 0.084 m, and by hybrid was 0.078 m. This means that the distance measurements by VLC were more spread out as compared to BLE and hybrid. This is because the maximum distance estimated by VLC can be as large as the diameter of the coverage area of the LED. The maximum error for the BLE estimated positions was 0.41 m for this environment, which was smaller than the diameter of the coverage area of the LED. This was caused by the fact that Bluetooth RSS-based systems work very well in small areas due to the shorter distance that the Bluetooth signal needs to travel to reach the receiver, leading to better positioning accuracy. Moreover, because multilateration takes three distances from the Bluetooth system and the $d_{4}$ is also estimated using the Bluetooth system, the hybrid multilateration method is very dependent on the accuracy of the Bluetooth estimation. As shown in Table 7, these results suggest that the VLC method tended to estimate distances in a further position than estimated by BLE and hybrid methods in the smaller space. However, the confidence interval for all three methods was relatively narrow, indicating a high degree of confidence in the estimated distances and satisfactory overall positioning accuracy.

### 6.2. Second Experiment Environment

We decided to extend the experiment into a larger scene to examine it further under more realistic conditions. Similar to the first experiment, 52 positions were marked to be estimated in a larger room where the same environment was duplicated to ensure the experiment had the same parameters and setup in regards to LED heights, beacon placement, and physical obstacles. The only difference was the size of the room, which was 6.7 × 5.4 × 3 m^3^. Similar to the first experiment, each of the 52 positions had 10 readings for Bluetooth and was under the coverage of the LED.

Following the same steps as in the first experiment, the positions to be estimated were marked on the floor. Ten readings were taken and averaged for each position. This was to ensure that any change in the vertical position or height of the device at the time of the reading did not affect positioning accuracy. However, results from the Bluetooth system showed lower accuracy in the larger space. Most of the positions were picked up outside of the coverage area of the LED, even though they picked up the signal from the light source. Therefore, it assumed the position of the center of the LED whenever the value of $d_{4}$ was larger than the outer radius of the LED while picking up the LED ID, giving an error value of 0.5 m or less for each position. The results of the experiments are shown below in Table 9.

**Figure 19:**
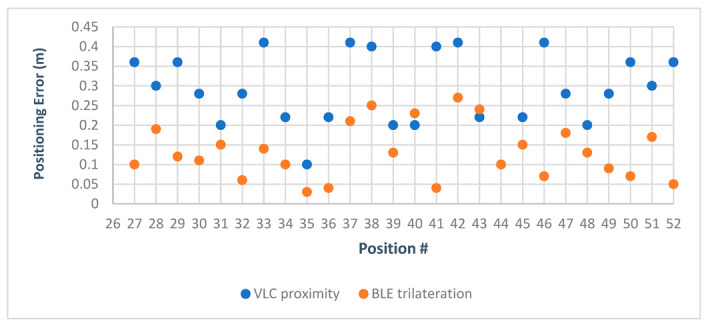
Positioning error under LED #1 for BLE and VLC in the second environment.

Figure 19 shows the difference in error between VLC and BLE under the first LED in the second environment. It is clear that BLE performed with a higher error rate than the error rate in the smaller environment. This can be attributed to the change in quality of the RSS signal in the larger area, and this is where the hybrid system came into play, choosing the optimal solution in 26 out of 26 cases under the first LED and giving an optimal solution percentage of 100%.

**Figure 20:**
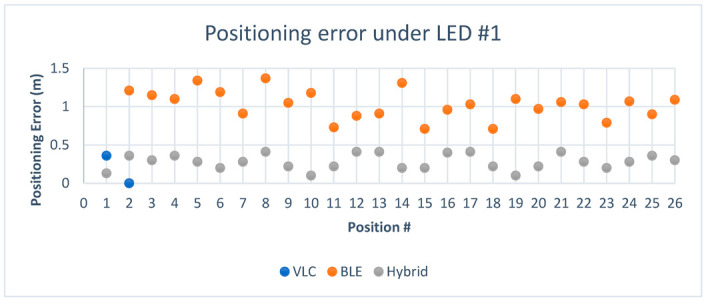
Positioning error under LED #1 for all technologies in the second environment.

Figure 20 shows the positioning error for the hybrid system in comparison to BLE and VLC. As shown, the hybrid system favored the VLC technology due to the higher error rate in BLE in the larger environment.

Unfortunately, the limitations of RSS-based techniques can be seen in the second experiment, as the error was significantly higher for BLE. This is because the signal strength of Bluetooth decreases as the distance between the transmitter and receiver increases. Fortunately, the hybrid system was designed to lean towards either the VLC or BLE systems based on the performance of both systems, comparing them against a dedicated threshold.

As shown by the graph below, the Bluetooth-estimated positions assumed the user stood in positions outside the coverage area of the LED, even though the device picked up the LED ID, indicating that it was indeed within the coverage area of that LED. Therefore, in these cases, the position was assumed to be equal to the value of the center of the LED, and in the rest of the cases, which were inside the coverage area of the LED but exceeded the value of the threshold for the inner radius, the position of the Bluetooth position was assumed to be similar to the first experiment.

As can be seen here, BLE-estimated positions were far from the center of the LED for both LEDs and the actual positions. This can be attributed to the variations in the RSSI signal resulting from the larger environment. VLC played a significant role in improving the positioning error for the radio frequency-based technology under these conditions. The hybrid system was designed to prioritize the system that performed optimally in a specific environment.

**Figure 21:**
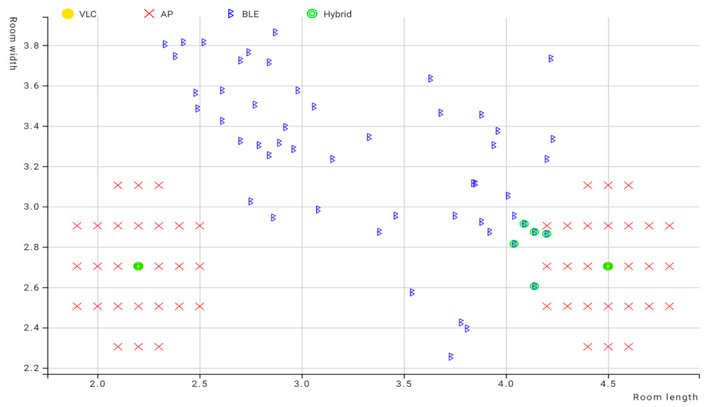
Actual vs. detected positions in the second experiment.

**Figure 22:**
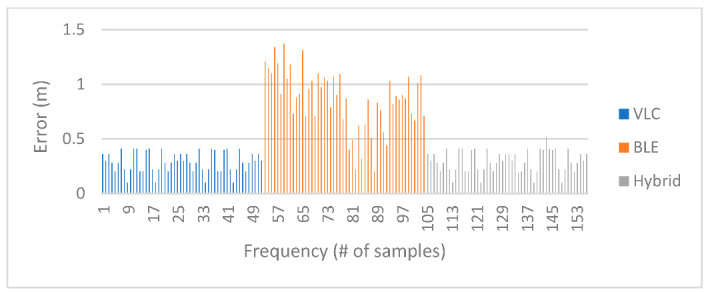
Error using different technologies in the second experiment.

Looking at Figure 21 and Figure 22, it can be deduced that the BLE readings in the new environment had higher error rates than in the smaller environment. After taking the BLE-estimated positions and comparing the values of $d_{4}$ against the threshold of the LED coverage, the positions that were estimated to be outside the coverage area of the LED while picking up the LED signal were assumed to have, as their initial value, the value of the center of the LED, such as positions (1–26) in Table 9. This improved the performance of the system, and it also demonstrated how VLC is able to support BLE-based positioning due to the LED’s limited coverage area. In regards to the hybrid algorithm in the second environment, only 2 out of 52 total positions did not achieve the optimal solution, achieving a percentage of optimal solutions for the hybrid system of 96.2% in the second environment. The hybrid mostly relied on VLC positioning since most of the BLE positions were estimated to be outside the coverage area of the LED, making their error rate higher than the value of the radius for the coverage area.

Table 10 shows that the VLC and hybrid positioning systems were more accurate and precise than the BLE system. The mean error for the VLC system was 0.29 m, which was lower than the BLE system’s mean error of 0.86 m. The hybrid system’s mean error was only slightly higher than the VLC system’s at 0.30 m. The minimum and maximum errors for the VLC and hybrid systems were also lower than those for the BLE system, indicating better accuracy and precision. The sum of errors for the VLC and hybrid systems was also much lower than the BLE system, indicating better overall performance. Additionally, the standard deviation and 95% confidence interval were also smaller for the VLC and hybrid systems compared to the BLE system, indicating greater precision in the measurements. Overall, based on the provided data, it appears that the VLC and hybrid positioning systems outperformed the BLE system in terms of accuracy and precision in the larger testing environment, with the hybrid system addressing the limitation of BLE’s accuracy, which is impacted by changes in the quality of RSS signals in the larger environment. The hybrid system appears to be a good compromise between the VLC and BLE systems, with a similar mean error and sum of errors as the VLC system but with the added benefit of BLE’s longer range.

These are the findings based on our observations:

- Within the first environment, the BLE system was able to achieve high positioning accuracy due to the higher quality of RSS signals, and the hybrid system achieved the optimal solution in 73.1% of the positions. The cases where the hybrid was not able to achieve the optimal solution were due to the quality of $d_{4}$, as it depended on the quality of the BLE-estimated position. Since most cases in this environment fell within the coverage area of the LED, the error in BLE positions was lower than the coverage area of the LED, which meant the hybrid did not lean towards the VLC in this area.
- In the second environment, the room was larger, which caused more fluctuations in RSS signals and less accurate BLE positioning. The hybrid system leaned towards VLC positioning because of the higher error value in positioning, which led to larger values of $d_{4}$. The hybrid system achieved the optimal solution in 96.2% of the positions in this area.
- The hybrid system worked by using either VLC proximity or BLE trilateration or a multilateration of both, depending on the value of the distance between the estimated position of the user’s device and the LED ($d_{4}$). This meant that in the worst case, accuracy would be limited by the value of the outer radius of the coverage area of the LED, which was 0.50 m.
- Taking the value of $d_{4}$ based on the BLE-estimated position gave the optimal solutions for more than 70% of cases in both environments. However, we believe that there is more to explore here regarding how to obtain the value of $d_{4}$ using different techniques, such as RSSI from the LED.
- Using VLC alone in the first environment would have achieved a higher error than using the hybrid, and using BLE alone in the second environment would have achieved a higher error than the hybrid system. The hybrid system helped lower the error whenever one of the systems gave less accurate results.

## 7. Evaluation of the Proposed System

To evaluate the quality of accuracy in the proposed system, we compared it against the existing literature for Bluetooth, VLC, and hybrid systems. Therefore, we calculated the positioning error using Euclidian distance, the average error, and the maximum and minimum error, as shown in Table 8 and Table 10, along with the Root Mean Square Error of each technology for each environment, as shown in Table 11.

In the first smaller environment, the performance of the Bluetooth system achieved highly accurate positioning, leading the hybrid system to achieve very similar accuracy since it performed better than the VLC system. However, in the second environment, the RMSE of the hybrid system was 0.31 m, showing how much VLC improved the performance of the Bluetooth system in the larger environment and achieving similar accuracy to the VLC system since it performed better in the second environment. This showed that the hybrid system was able to lean towards the system with higher accuracy depending on its performance and take advantage of it. 

Within the literature, various studies have suggested using VLC for positioning. Ref. [8] proposed a collaborative indoor visible light positioning (VLP) system that uses a BLE-improved spring model to enhance accuracy. This system can be combined with the fingerprinting technique without the need for additional sensors by utilizing neighboring mobile devices. The simulation results indicated an average accuracy of 6.0 cm [8]. Moreover, Zhang et al. [16] used VLC triangulation and achieved a precision of 95% within 17.25 cm with direct sunlight exposure and a precision of 95% within 11.2 cm with indirect sunlight exposure. Li et al. [17] achieved an error of less than 1 m in a large indoor space using VLC trilateration. Gu et al. [18] achieved a localization accuracy of 0.10–0.09 cm using VLC trilateration, with the Kalman filter and SIR filter improving the accuracy further. Zhao et al. [19] reported that their system achieved an average accuracy of 0.50, 0.50, and 1.60 m in three different test environments using LightPrint. Table 11 presents a comparison between this work and other literature in regards to design and accuracy. This work achieved a high level of accuracy with a minimum error of 0.03 m using Euclidean distance and an average error of 0.14–0.30 m for Euclidean distance in the smaller environment and the larger environment, respectively. It also achieved an error of 0.16 m using RMSE in the smaller environment and 0.31 m using RMSE in the larger environment. The maximum error was 0.52 m, which was measured in the larger environment, while the maximum error in the smaller environment was 0.41 m. Overall, the results suggest that the current work performed very well against the other systems evaluated in terms of accuracy, with a minimum error and a low average error.

Comparing this work to the literature helps us answer the research questions that motivated this research.

Q1. Does VLC proximity provide accurate results for indoor positioning?

**Figure 23:**
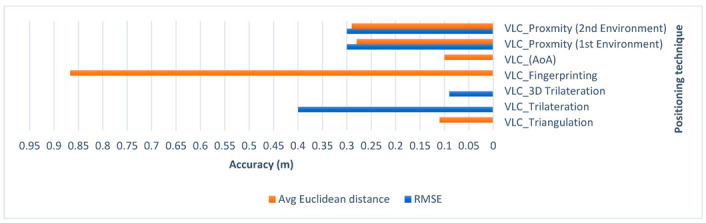
Comparison of the localization accuracy of different VLC systems [16,17,18,19,20].

A1. Despite the simplicity of the proximity algorithm, the VLC system achieved accurate results for both experiments. As shown in Figure 23, the system obtained a mean error of 0.28 m in the first environment, a minimum error of 0.10 m, and a maximum error of 0.46 m. For the second environment, it achieved a mean error of 0.29 m, a minimum of 0.10 m, and a maximum of 0.41 m. An overall RMSE of 0.30 m was found for both environments. This showed that proximity can achieve accurate results for indoor positioning in comparison to other complex approaches mentioned in Table 12, such as triangulation [16], where the device needs at least three signals to achieve similar results. The same was true for the system proposed by Li et al. in [17], where they achieved an RMSE of 0.40 m for a VLC trilateration/multilateration-based system, while our study achieved an RMSE of 0.30 m using proximity alone. In [18], Gu et al. proposed three-dimensional positioning using VLC trilateration with two filter improvements to achieve an RMSE of 0.09 m, which indicated that the use of trilateration alone can be improved with the use of filtering techniques. Other papers addressed the accuracy issue by using different approaches, such as fingerprinting in [19], where they used Light Intensity Field maps to match the LightPrint. They achieved an accuracy of 0.50 to 1.60 m, although their system demanded huge computational complexity due to a curve-surface matching problem. Another approach, AoA, was used in [20], where they achieved an error rate of around 0.10 m despite many challenges and limitations in using this technique, such as camera resolution affecting the quality of the signals and the image quality requiring filtering. However, this does not mean VLC proximity is the most accurate technique of VLC-based positioning; rather, we showed that despite the simplicity and low complexity of this approach, it is still able to achieve accurate indoor positioning since the error rate is limited by the coverage area of the LED used in the experiment and it is not subject to signal interference like radio-based technologies, enabling it to achieve real-time positioning that could be helpful for many applications, such as exploring exhibits and galleries, or for manufacturing purposes and goods placement. Overall, based on our observations, we suggest that accuracy could be improved by combining VLC proximity with other techniques or technologies.

Q2. Does Bluetooth trilateration provide accurate results for indoor positioning?

**Figure 24:**
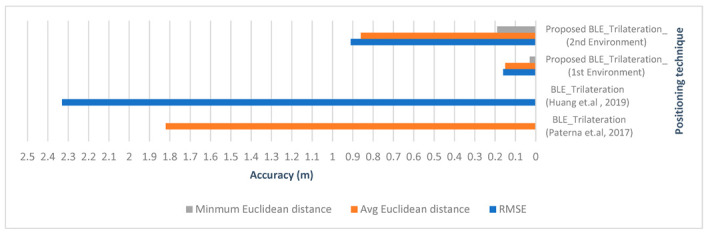
Comparison of the localization accuracy of different BLE systems [23,28].

A2. As proved by the literature and shown in Figure 24, BLE trilateration does provide accurate results for indoor positioning. It was shown in [23] by Paterna et al. that a system that uses Bluetooth trilateration with frequency diversity and Kalman filtering was able to achieve an average error of 1.82 m in a 9.19 m × 6.18 m room. Moreover, in [28], Huang et al. achieved an accuracy of 0.76 m using a hybrid method of trilateration and dead reckoning.

In this work, the Bluetooth system achieved accurate results for both environments, with a mean of 0.15 m and 0.86 m for the first and second environments, respectively. It achieved a minimum of 0.03 m of positioning error in the first environment. However, some limitations in this technology became apparent in the second environment, as the quality of the positioning was affected by the change in RSS. Even though the BLE system achieved a minimum error of 0.19 m in the second environment, the maximum was 1.37 m, which was still considered good for indoor positioning but was not as accurate as the positions obtained in the smaller area. The use of hybrid positioning shows promise in these situations. Looking at both the literature and this work, we can conclude that although Bluetooth trilateration achieves accurate results, in almost all of the mentioned studies there was an improvement factor used to achieve better accuracy, whether a filtering technique or a hybrid with another positioning technique, showing that Bluetooth trilateration can work well with other positioning techniques and technologies.

Q3. Does combining VLC proximity with RSS-based Bluetooth trilateration in a hybrid system achieve accurate results for indoor positioning?

**Figure 25:**
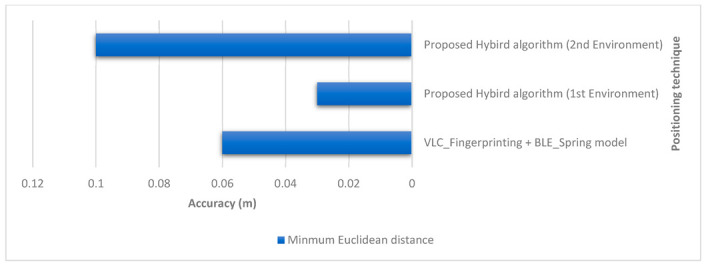
Comparison of positioning accuracy of hybrid systems [8].

A3. As shown in Table 11 and Figure 25, the results suggested that the current work performed very well against the other systems evaluated in terms of accuracy, with a low minimum error and a low average error. In the first experiment, even though the BLE error rates were low, VLC still managed to lower the error by multilateration in some cases. Additionally, the hybrid algorithm helped to improve the error in BLE positioning in the larger environment by limiting the error within the coverage area of the LED whenever the user was assumed to be outside of it. We can deduce from the experiment that the hybrid system provided accurate results for indoor positioning. Using both had the advantage of overcoming the limited coverage area of the LED for VLC proximity when the BLE system was performing with a higher error margin than that coverage area. Taking advantage of the BLE system whenever the quality of the RSS signals from the Bluetooth beacons required it, we achieved high accuracy using trilateration. The minimum error for the first environment of the hybrid system was 0.03 m, while for the second environment it was 0.10 m. The mean errors for the first and second environments were 0.14 m and 0.30 m, respectively. The RMSEs for the first and second environments were 0.16 m and 0.31 m, respectively. As suggested in [8], the collaboration between a VLC positioning system and a Bluetooth-based system improved the positioning error, achieving an accuracy of 0.06 m. However, computational complexity is a general setback of the fingerprinting technique in almost any system [4]. Overall, we deduced from the literature that the combination of positioning techniques and technologies in hybrid systems shows promising results for indoor positioning.

## 8. Conclusions

The main aim of this paper was to explore and navigate the gaps within indoor positioning, especially within the field of VLC, which is a promising technology with high accuracy and low interference. It has the potential to revolutionize indoor navigation. This paper investigated a novel hybrid indoor positioning system that uses Visible Light Communication (VLC) proximity with Bluetooth trilateration technology to offer a robust and accurate solution for indoor positioning. By combining the simplicity of VLC proximity with the complexity of Bluetooth trilateration, the system provided a highly accurate and reliable indoor positioning solution.

In conclusion, this paper presented a hybrid methodology to calculate a user’s position in an indoor environment using VLC proximity, Bluetooth trilateration, and a hybrid of both methods. Carrying out the experiment in two different-sized environments gave an advantage to either the BLE or VLC technology in terms of accurate results; therefore, the hybrid method was presented in order to take advantage of both technologies as best as possible.

In the future, we would like to explore the system in more environments and with different settings with respect to the equipment to investigate the effects of these changes on the system’s performance. Regarding the Bluetooth system, we may experiment with altering the height and placement of the beacons to examine their impact on the RSS signals, and we may also explore RSS filtering techniques, such as Kalman filtering [23], to see how they affect the system’s accuracy. For the VLC system, we could investigate the use of RSS measurement techniques, such as a luxmeter, to determine the precise position between the user’s location and the VLC LED.

## Figures and Tables

**Table 1:** Comparison between VLC-based systems.

System	Technology	Modulation Technique	Receiver Type	Positioning Technique	Real-Time Ability	Accuracy (m)
[8]	BLE + VLC	Not mentioned	Smart Mobile	Fingerprinting + Spring model	Yes	0.06 m
[16]	VLC	On–Off Keying (OOK)	Optical Receiver	Triangulation	No	0.17–0.11 m
[17]	VLC	Binary Frequency Shift Keying (BFSK)	Smart Mobile	Trilateration	No	0.4 m
[18]	VLC	On–Off Keying (OOK)	Photodiodes	Trilateration	No	~0.10–0.09 m
[19]	VLC	-	Smart Mobile	Fingerprinting	No	0.50 m, 0.50 m 1.60 m
[20]	VLC	On–Off Keying (OOK)	Smart Mobile	Angel of Arrival (AoA)	No	~0.1 m

**Table 2:** The positioning scenarios of the proposed system.

Case	Condition Statement	Action
If the distance between the BLE estimated position (xb, yb) and the LED (xi, yi) is within the inner radius	$d_{4}$ ≤ $R_{in}$ → Multilateration	Use Multilateration of all four distances
If the distance between the BLE estimated position (xb, yb) and the LED (xi, yi) is smaller than the outer radius but larger than the inner radius	$R_{in}$ < $d_{4}$ ≤ $R_{out}$ → BLE	Use the BLE estimated position
If the distance between the BLE estimated position (xb, yb) and the LED (xi, yi) is larger than the outer radius, meaning that it is outside the coverage area of the LED	$d_{4}$ > $R_{out}$ → VLC	Use the VLC estimated position

**Table 3:** Environment parameters of the first space.

Parameters	Value
Room size	3.7 × 3.3 × 3 m^3^
Number of LEDs	2
Coordinates of LEDs	A (1.2, 1.7) B (2.5, 1.7)
LED height	1.5 m
Number of beacons	3
Coordinates of beacons	C (0.5, 0.5) D (3.2, 0.5) E (1.9, 2.8)

**Table 4:** Environment parameters of the second space.

Parameters	Value
Room size	6.7 × 5.4 × 3 m^3^
Number of LEDs	2
Coordinates of LEDs	A (2.2, 2.7) B (4.5, 2.7)
LED height	1.5 m
Number of beacons	3
Coordinates of beacons	C (0.6, 4.8) D (6.1, 4.8) E (3.35, 0.6)

**Table 5:** LED specifications.

LED Specifications	
Standard	IEEE 802.15
Height	1.5 m
Coverage Radius	0.50 m
Power	14.8 W
Voltage	234.7 V AC

**Table 6:** Beacon specifications.

Range	~70 m
Broadcasting power	4 dBm
Advertising interval	200 ms
Supported protocols	iBeacon, Eddystone

**Table 7:** Positioning results for the first LED.

#	LED #0x270f	D4 Value	Error		
	Actual Position		VLC	BLE	Hybrid
1	0.9, 1.5	0.42	0.36	0.13	0.13
2	0.9, 1.7	0.19	0.30	0.15	0.15
3	0.9, 1.9	0.19	0.36	0.31	0.31
4	1.0, 1.5	0.25	0.46	0.06	0.06
5	1.0, 1.7	0.11	0.20	0.09	0.08
6	1.0, 1.9	0.20	0.28	0.09	0.09
7	1.1, 1.3	0.36	0.41	0.12	0.12
8	1.1, 1.5	0.09	0.22	0.15	0.19
9	1.1, 1.7	0.12	0.10	0.18	0.16
10	1.1, 1.9	0.36	0.22	0.15	0.15
11	1.1, 2.1	0.18	0.41	0.23	0.23
12	1.2, 1.3	0.45	0.40	0.09	0.09
13	1.2, 1.5	0.25	0.20	0.22	0.22
14	1.2, 1.9	0.23	0.23	0.20	0.20
15	1.2, 2.1	0.58	0.40	0.41	0.41
16	1.3, 1.3	0.39	0.41	0.04	0.04
17	1.3, 1.5	0.23	0.22	0.13	0.13
18	1.3, 1.7	0.29	0.10	0.19	0.19
19	1.3, 1.9	0.32	0.22	0.14	0.14
20	1.3, 2.1	0.45	0.41	0.24	0.24
21	1.4, 1.5	0.26	0.28	0.04	0.04
22	1.4, 1.7	0.27	0.20	0.09	0.09
23	1.4, 1.9	0.45	0.28	0.22	0.22
24	1.5, 1.5	0.45	0.36	0.10	0.10
25	1.5, 1.7	0.24	0.30	0.11	0.11
26	1.5, 1.9	0.47	0.36	0.11	0.11

**Table 8:** Error using Euclidian distance in the first experiment.

	VLC	BLE	Hybrid
Mean	0.28 m	0.15 m	0.14 m
Minimum	0.10 m	0.03 m	0.03 m
Maximum	0.46 m	0.41 m	0.41 m
Total distance (Sum)	14.73 m	7.85 m	7.46 m
Standard deviation	0.10 m	0.084 m	0.078 m
Confidence interval 95%	0.027 m	0.023 m	0.021 m

**Table 9:** Distance error for each position for the first LED.

#	LED #0x270f	D4 Value	Error		
	Actual Position		VLC	BLE	Hybrid
1	1.9, 2.5	0.90	0.36	1	1.9, 2.5
2	1.9, 2.7	1.05	0.30	2	1.9, 2.7
3	1.9, 2.9	1.16	0.36	3	1.9, 2.9
4	2.0, 2.5	1.11	0.28	4	2.0, 2.5
5	2.0, 2.7	1.13	0.20	5	2.0, 2.7
6	2.0, 2.9	0.84	0.28	6	2.0, 2.9
7	2.1, 2.3	0.98	0.41	7	2.1, 2.3
8	2.1, 2.5	0.83	0.22	8	2.1, 2.5
9	2.1, 2.7	1.14	0.10	9	2.1, 2.7
10	2.1, 2.9	0.80	0.22	10	2.1, 2.9
11	2.1, 3.1	0.96	0.41	11	2.1, 3.1
12	2.2, 2.3	0.64	0.41	12	2.2, 2.3
13	2.2, 2.5	1.17	0.20	13	2.2, 2.5
14	2.2, 2.9	0.84	0.20	14	2.2, 2.9
15	2.2, 3.1	1.09	0.40	15	2.2, 3.1
16	2.3, 2.3	0.92	0.41	16	2.3, 2.3
17	2.3, 2.5	0.70	0.22	17	2.3, 2.5
18	2.3, 2.7	1.17	0.10	18	2.3, 2.7
19	2.3, 2.9	1.20	0.22	19	2.3, 2.9
20	2.3, 3.1	1.30	0.41	20	2.3, 3.1
21	2.4, 2.5	1.00	0.28	21	2.4, 2.5
22	2.4, 2.7	0.83	0.20	22	2.4, 2.7
23	2.4, 2.9	1.34	0.28	23	2.4, 2.9
24	2.5, 2.5	0.92	0.36	24	2.5, 2.5
25	2.5, 2.7	1.19	0.30	25	2.5, 2.7
26	2.5, 2.9	0.96	0.36	26	2.5, 2.9

**Table 10:** Error using Euclidian distance.

	VLC	BLE	Hybrid
Mean	0.29 m	0.86 m	0.30 m
Minimum	0.10 m	0.19 m	0.10 m
Maximum	0.41 m	1.37 m	0.52 m
Sum	14.97 m	44.83 m	15.39 m
Standard deviation	0.09 m	0.28 m	0.10 m
Confidence interval 95%	0.025 m	0.076 m	0.027 m

**Table 11:** RMSE Error for all technologies in both testing environments.

	VLC	BLE	Hybrid
RMSE for the 1st Environment	0.30 m	0.16 m	0.16 m
RMSE for the 2nd Environment	0.30 m	0.91 m	0.31 m

**Table 12:** Evaluation of the proposed system.

System	Technology	Positioning Technique	Deployment Area	Deployment Density	Accuracy (m)	Evaluation Method	Type
[8]	BLE + VLC	Fingerprinting + Spring model	5 × 5 × 3 m^3^	4 LEDs	0.06 m	Euclidean distance	Minimum Error
[16]	VLC	Triangulation	6 × 6 × 4 m^3^	4 LEDs	0.17–0.11 m	Euclidean distance	Average Error
[17]	VLC	Trilateration	(a) 5 × 8 m^2^ (b) 2 × 12 m^2^ (c) 3.5 × 6.5 m^2^	5 LEDs	0.40 m	RMSE	Average Error
[18]	VLC	3D Trilateration	6 × 6 × 4.2 m^3^	4 LEDs	~0.10–0.09 m	RMSE	Average Error
[19]	VLC	Fingerprinting	(a) 625 m^3^ (b) 148 m^3^ (c) 260 m^3^	130 LEDs 38 LEDs 20 LEDs	0.50 m 0.50 m 1.60 m	Euclidean distance	Average Error
[20]	VLC	Angel of Arrival (AoA)	0.711 × 0.737 m^2^	5 LEDs	~0.10 m	Euclidean distance	Average Error
This work	VLC + BLE	Proximity + Trilateration	(a) 3.7 × 3.3 × 3 m^3^ (b) 6.7 × 5.4 × 3 m^3^	2 LEDs	0.03 m 0.52 m 0.14–0.30 m 0.16–0.31 m	Euclidean distance + RMSE	Minimum Error Maximum Error Average Error ED Average Error RMSE

## Data Availability

The data presented in this study are available in the Supplementary Material.

## Supplementary Materials

The following supporting information can be downloaded at: https://www.mdpi.com/article/10.3390/s23167199/s1.
