# Supplementary material for: A Hybrid Indoor Positioning System Based on Visible Light Communication and Bluetooth RSS Trilateration
Source: Sensors (Basel). 2023 Aug 16;23(16):7199. doi: 10.3390/s23167199 (PMC10459576; doi:10.3390/s23167199)
Supplement: Supplementary file 1 [file sensors-23-07199-s001.zip › sensors-2452661-supplementary.pdf]

First Experiment

First LED

#	LED #0x270f	D4 value	Error		
	**Actual position**		**VLC**	**BLE**	**Hybrid**
**1**	**0.9, 1.5**	0.42	0.36	0.13	0.13
**2**	**0.9, 1.7**	0.19	0.30	0.15	0.15
**3**	**0.9, 1.9**	0.19	0.36	0.31	0.31
**4**	**1.0, 1.5**	0.25	0.46	0.06	0.06
**5**	**1.0, 1.7**	0.11	0.20	0.09	0.08
**6**	**1.0, 1.9**	0.20	0.28	0.09	0.09
**7**	**1.1, 1.3**	0.36	0.41	0.12	0.12
**8**	**1.1, 1.5**	0.09	0.22	0.15	0.19
**9**	**1.1, 1.7**	0.12	0.10	0.18	0.16
**10**	**1.1, 1.9**	0.36	0.22	0.15	0.15
**11**	**1.1, 2.1**	0.18	0.41	0.23	0.23
**12**	**1.2, 1.3**	0.45	0.40	0.09	0.09
**13**	**1.2, 1.5**	0.25	0.20	0.22	0.22
**14**	**1.2, 1.9**	0.23	0.23	0.20	0.20
**15**	**1.2, 2.1**	0.58	0.40	0.41	0.41
**16**	**1.3, 1.3**	0.39	0.41	0.04	0.04
**17**	**1.3, 1.5**	0.23	0.22	0.13	0.13
**18**	**1.3, 1.7**	0.29	0.10	0.19	0.19
**19**	**1.3, 1.9**	0.32	0.22	0.14	0.14
**20**	**1.3, 2.1**	0.45	0.41	0.24	0.24
**21**	**1.4, 1.5**	0.26	0.28	0.04	0.04
**22**	**1.4, 1.7**	0.27	0.20	0.09	0.09
**23**	**1.4, 1.9**	0.45	0.28	0.22	0.22
**24**	**1.5, 1.5**	0.45	0.36	0.10	0.10
**25**	**1.5, 1.7**	0.24	0.30	0.11	0.11
**26**	**1.5, 1.9**	0.47	0.36	0.11	0.11

Second LED

#	LED #0xa7d5	D4 value	Error

	Actual Position		VLC	BLE	Hybrid
**1**	**2.2, 1.5**	0.44	0.36	0.10	0.10
**2**	**2.2, 1.7**	0.41	0.30	0.19	0.19
**3**	**2.2, 1.9**	0.48	0.36	0.12	0.12
**4**	**2.3, 1.5**	0.34	0.28	0.11	0.11
**5**	**2.3, 1.7**	0.27	0.20	0.15	0.15
**6**	**2.3, 1.9**	0.35	0.28	0.06	0.06
**7**	**2.4, 1.3**	0.51	0.41	0.14	0.14
**8**	**2.4, 1.5**	0.30	0.22	0.10	0.10
**9**	**2.4, 1.7**	0.12	0.10	0.03	0.03
**10**	**2.4, 1.9**	0.19	0.22	0.04	0.04
**11**	**2.4, 2.1**	0.21	0.41	0.21	0.21
**12**	**2.5, 1.3**	0.28	0.40	0.25	0.25
**13**	**2.5, 1.5**	0.31	0.20	0.13	0.13
**14**	**2.5, 1.9**	0.10	0.20	0.23	0.27
**15**	**2.5, 2.1**	0.44	0.40	0.04	0.04
**16**	**2.6, 1.3**	0.38	0.41	0.27	0.27
**17**	**2.6, 1.5**	0.06	0.22	0.24	0.23
**18**	**2.6, 1.7**	0.11	0.10	0.10	0.09
**19**	**2.6, 1.9**	0.13	0.22	0.15	0.16
**20**	**2.6, 2.1**	0.34	0.41	0.07	0.07
**21**	**2.7, 1.5**	0.27	0.28	0.18	0.18
**22**	**2.7, 1.7**	0.11	0.20	0.13	0.14
**23**	**2.7, 1.9**	0.31	0.28	0.09	0.09
**24**	**2.8, 1.5**	0.30	0.36	0.07	0.07
**25**	**2.8, 1.7**	0.30	0.30	0.17	0.17
**26**	**2.8, 1.9**	0.38	0.36	0.05	0.05

SECOND EXPERIMNET

First led

#	LED #0x270f	D4 value	Error		
	Actual Position		VLC	BLE	Hybrid
**1**	**1.9, 2.5**	0.90	0.36	1.21	0.36

**2**	**1.9, 2.7**	1.05	0.30	1.15	0.30
**3**	**1.9, 2.9**	1.16	0.36	1.10	0.36
**4**	**2.0, 2.5**	1.11	0.28	1.34	0.28
**5**	**2.0, 2.7**	1.13	0.20	1.19	0.20
**6**	**2.0, 2.9**	0.84	0.28	0.91	0.28
**7**	**2.1, 2.3**	0.98	0.41	1.37	0.41
**8**	**2.1, 2.5**	0.83	0.22	1.05	0.22
**9**	**2.1, 2.7**	1.14	0.10	1.18	0.10
**10**	**2.1, 2.9**	0.80	0.22	0.73	0.22
**11**	**2.1, 3.1**	0.96	0.41	0.88	0.41
**12**	**2.2, 2.3**	0.64	0.41	0.91	0.41
**13**	**2.2, 2.5**	1.17	0.20	1.31	0.20
**14**	**2.2, 2.9**	0.84	0.20	0.71	0.20
**15**	**2.2, 3.1**	1.09	0.40	0.96	0.40
**16**	**2.3, 2.3**	0.92	0.41	1.03	0.41
**17**	**2.3, 2.5**	0.70	0.22	0.71	0.22
**18**	**2.3, 2.7**	1.17	0.10	1.10	0.10
**19**	**2.3, 2.9**	1.20	0.22	0.97	0.22
**20**	**2.3, 3.1**	1.30	0.41	1.06	0.41
**21**	**2.4, 2.5**	1.00	0.28	1.03	0.28
**22**	**2.4, 2.7**	0.83	0.20	0.79	0.20
**23**	**2.4, 2.9**	1.34	0.28	1.07	0.28
**24**	**2.5, 2.5**	0.92	0.36	0.90	0.36
**25**	**2.5, 2.7**	1.19	0.30	1.09	0.30
**26**	**2.5, 2.9**	0.96	0.36	0.68	0.36

Second LED

#	LED #0xa7d5	D4 value	Error		
	**Actual Position**		**VLC**	**BLE**	**Hybrid**
**1**	**4.2, 2.5**	0.36	0.30	0.87	0.36
**2**	**4.2, 2.7**	0.30	0.36	0.40	0.30
**3**	**4.2, 2.9**	0.36	0.28	0.49	0.36
**4**	**4.3, 2.5**	0.28	0.20	0.22	0.19
**5**	**4.3, 2.7**	0.20	0.28	0.62	0.20
**6**	**4.3, 2.9**	0.28	0.41	0.32	0.28
**7**	**4.4, 2.3**	0.41	0.22	0.63	0.41

**8**	**4.4, 2.5**	0.22	0.10	0.86	0.22
**9**	**4.4, 2.7**	0.10	0.22	0.51	0.10
**10**	**4.4, 2.9**	0.22	0.41	0.20	0.20
**11**	**4.4, 3.1**	0.41	0.40	0.83	0.41
**12**	**4.5, 2.3**	0.40	0.20	0.76	0.40
**13**	**4.5, 2.5**	0.20	0.20	0.56	0.52
**14**	**4.5, 2.9**	0.20	0.40	0.44	0.41
**15**	**4.5, 3.1**	0.40	0.41	1.03	0.40
**16**	**4.6, 2.3**	0.41	0.22	0.82	0.41
**17**	**4.6, 2.5**	0.22	0.10	0.89	0.22
**18**	**4.6, 2.7**	0.10	0.22	0.86	0.10
**19**	**4.6, 2.9**	0.22	0.41	0.90	0.22
**20**	**4.6, 3.1**	0.41	0.28	0.87	0.41
**21**	**4.7, 2.5**	0.28	0.20	1.07	0.28
**22**	**4.7, 2.7**	0.20	0.28	0.73	0.20
**23**	**4.7, 2.9**	0.28	0.36	0.67	0.28
**24**	**4.8, 2.5**	0.36	0.30	1.01	0.36
**25**	**4.8, 2.7**	0.30	0.36	1.08	0.30
**26**	**4.8, 2.9**	0.36	0.30	0.71	0.36
